# A retrospective, deformable registration analysis of the impact of PET-CT planning on patterns of failure in stereotactic body radiation therapy for recurrent head and neck cancer

**DOI:** 10.1186/1758-3284-4-12

**Published:** 2012-04-19

**Authors:** Kyle Wang, Dwight E Heron, John C Flickinger, Jean-Claude M Rwigema, Robert L Ferris, Gregory J Kubicek, James P Ohr, Annette E Quinn, Cihat Ozhasoglu, Barton F Branstetter

**Affiliations:** 1 Departments of Radiation Oncology, University of Pittsburgh Cancer Institute, Pittsburgh, PA, USA; 2 Otolaryngology, University of Pittsburgh Cancer Institute, Pittsburgh, PA, USA; 3 Neurological Surgery, University of Pittsburgh Cancer Institute, Pittsburgh, PA, USA; 4 Medical Oncology, University of Pittsburgh Cancer Institute, Pittsburgh, PA, USA; 5 Radiology, University of Pittsburgh Cancer Institute,, Pittsburgh, PA, USA

**Keywords:** Stereotactic body radiotherapy, Recurrent head & neck cancer, Salvage, PET-CT, Patterns of failure

## Abstract

**Background:**

Stereotactic body radiation therapy (SBRT) has seen increasing use as a salvage strategy for selected patients with recurrent, previously-irradiated squamous cell carcinoma of the head and neck (rSCCHN). PET-CT may be advantageous for tumor delineation and evaluation of treatment failures in SBRT. We analyzed the patterns of failure following SBRT for rSCCHN and assessed the impact of PET-CT treatment planning on these patterns of failure.

**Methods:**

We retrospectively reviewed 96 patients with rSCCHN treated with SBRT. Seven patients (7%) were treated after surgical resection of rSCCHN and 89 patients (93%) were treated definitively. PET-CT treatment planning was used for 45 patients whereas non-PET-CT planning was used for 51 patients. Categories of failure were assigned by comparing recurrences on post-treatment scans to the planning target volume (PTV) from planning scans using the deformable registration function of VelocityAI™. Failures were defined: In-field (>75% inside PTV), Overlap (20-75% inside PTV), Marginal (<20% inside PTV but closest edge within 1cm of PTV), or Regional/Distant (more than 1cm from PTV).

**Results:**

Median follow-up was 7.4 months (range, 2.6–52 months). Of 96 patients, 47 (49%) developed post-SBRT failure. Failure distribution was: In-field–12.3%, Overlap–24.6%, Marginal–36.8%, Regional/Distant–26.3%. There was a significant improvement in overall failure-free survival (log rank p = 0.037) and combined Overlap/Marginal failure-free survival (log rank p = 0.037) for those receiving PET-CT planning vs. non-PET-CT planning in the overall cohort (n = 96). Analysis of the definitive SBRT subgroup (n = 89) increased the significance of these findings (overall failure: p = 0.008, Overlap/Marginal failure: p = 0.009). There were no significant differences in age, gender, time from prior radiation, dose, use of cetuximab with SBRT, tumor differentiation, and tumor volume between the PET-CT and non-PET-CT groups.

**Conclusions:**

Most failures after SBRT treatment for rSCCHN were near misses, i.e. Overlap/Marginal failures (61.4%), suggesting an opportunity to improve outcomes with more sensitive imaging. PET-CT treatment planning showed the lowest rate of overall and near miss failures and is beneficial for SBRT treatment planning.

## Background

Salvage options are limited for recurrent, previously-irradiated squamous cell carcinoma of the head and neck (rSCCHN), resulting in an overall poor prognosis with a reported median survival of less than one year [1]. For rSCCHN, surgical salvage remains the gold standard but is only possible in approximately 20% of patients [2,3]. Re-irradiation as a salvage strategy is frequently limited by the normal tissue tolerance of previously-irradiated head and neck structures such as the brainstem, mandible, and spinal cord, but has resulted in a median survival of 9–14 months [4-8]. Stereotactic body radiation therapy (SBRT) has seen increasing use as a salvage strategy for inoperable cancers due to its ability to deliver potent doses of radiation to focal areas with sub-millimeter accuracy, minimizing injury to previously-irradiated tissues and shortening treatment courses. Studies on SBRT for rSCCHN at the University of Pittsburgh Cancer Institute (UPCI) and other institutions have demonstrated control rates comparable to conventional radiotherapy but with fewer toxicities [9-12].

In patients previously treated with surgery, chemotherapy, and/or radiation, delineation of recurrences can be challenging. Treatment planning involves manual delineation of tumors, and can be performed on an integrated PET-CT scan, a PET-aided CT scan, or a CT scan alone. PET-CT has been shown in primary disease to be more sensitive than CT, PET, or PET-aided scans [13]. Whereas in primary SCCHN it is accepted practice to contour regions of lymphatic drainage and sizeable margins around the gross tumor volume (GTV), there is no current standard regarding the use of such margins for recurrent tumors [8]. Current studies on SBRT for rSCCHN, however, have used small or no margins to minimize toxicity [9,10,12,14]. Hence, PET-CT is ideal for delineation in SBRT, whose sharp dose fall-off makes accurate targeting essential. There is yet no data showing that PET-CT is better than non-PET-CT in SBRT treatment planning, judged best by treatment outcome and patterns of failure. We hypothesized that PET-CT planning would lead to a reduction in overall and in particular “near miss” failures, i.e. failures at the PTV border.

The most critical question in any analysis of radiation therapy failures is “Was the tumor missed?” When recurrences develop close to SBRT targets (with rapid dose fall-off outside targets), this question can be very difficult to answer because follow-up scans are usually done with patients in a different position from when they were treated. To overcome this obstacle and analyze patterns of failure, we used the deformable registration function of the VelocityAI^TM^ [Velocity Medical Systems, Atlanta, GA] software program to compare the location of recurrences on post-treatment scans to the PTV from planning scans. Failures within the PTV may imply resistant tumors or insufficient dose, whereas failures near PTV borders could represent insufficient margins or failure of imaging to detect tumor edges. Few studies exist on patterns of failure after SBRT and the current study is unique in the degree of accuracy we attempt to achieve in using deformable registration to identify the location of recurrences.

## Methods

### Study design and patient selection

A retrospective cohort study was performed drawing upon the largest published series from the UPCI experience in treating rSCCHN with SBRT. All patients signed a written informed consent for treatment and the study was approved by the institutional review board. Data were de-identified to meet the Health Insurance Portability and Accountability guidelines. All patients had previously-irradiated, recurrent SCCHN and were originally treated with surgery, chemotherapy, and/or external beam radiation for their primary malignancies. Patients were 18 years of age or older, had a Karnofsky performance status (KPS) of 50 or greater, and received no chemotherapy or radiation for at least one month before entry into the study. Patients were excluded if SBRT was part of treatment for a primary tumor or if they had not received prior radiation therapy.

Between June 2005 and November 2009, 111 patients with recurrent, previously-irradiated SCCHN meeting these criteria received either Cyberknife^TM^ or Trilogy^TM^ SBRT, described in our prior reports [15,16]. Fifteen patients had no post-treatment PET-CT scans and were excluded from the analysis. Of 96 final patients, 40 (42%) had distant metastases or untreated locoregional disease and were treated palliatively. Fifty-six patients (58%) had no other tumors and were treated with curative salvage intent. Seven patients (7%) were treated with adjuvant SBRT after surgical resection of rSCCHN and 89 patients (93%) were treated definitively with SBRT. All primary tumors were confirmed to be squamous cell carcinoma on pathology reports. Biopsies of recurrent lesions were not routinely performed if there was sufficient clinical suspicion of recurrence of the original tumor based on imaging, history, or physical examination.

### Treatment planning and delivery

Treatment plans for patients in this study were created using an integrated PET-CT scan, a PET-aided CT (PET-CT from a different date fused to a CT with rigid registration), or a CT-only scan. The planning CT was acquired with 1.25–mm thick slices in the target area. Physicians contoured the spinal cord and other critical structures on the planning CT. The GTV was contoured by visual inspection and treated without a margin (GTV = PTV).

Treatment plans were developed based on tumor geometry and nearby critical structures. All patients received five fractions and most (89%) received 40–50 Gy. Dose limits for normal structures were selected based on prior irradiation and the radiation oncologist’s clinical judgment. Dose limits were: 8 Gy (1 fraction) for spinal cord, 9 Gy for brainstem, 20 Gy for brain, 10 Gy for retina, optic nerves, and optic chiasm, 6 Gy for lens, 20 Gy for carotid artery, and 20 Gy for esophagus and larynx. Dose-volume histograms for at-risk structures were used to verify these limits. Phantom dose measurements were used for quality assurance. Skull or cervical spine tracking was used to localize lesions with a 1–mm spatial accuracy [17].

Before treatment, patients were placed on the treatment table with an immobilization device then fitted with a personalized thermoplastic facemask secured to the headrest. Near-real-time digital x-rays or cone-beam CT images were obtained during treatment to establish and verify target locations. Treatments were administered every other day. All patients were seen one to three months after treatment and received a PET-CT, CT, or MRI as part of their follow-up.

### Assessment of patterns of failure

Patterns of failure were analyzed using VelocityAI^TM^, which uses a modified B-spline deformable registration algorithm. Testing of this algorithm demonstrated a mean error of 1–2 mm for noise-free images [18,19]. Treatment response was assessed using the RECIST (Response Evaluation Criteria for Solid Tumors) criteria [20]. Failure was defined as initial disease progression or complete response/partial response/stable disease followed later by progressive disease. Development of regional/distant disease (defined below) was also considered failure if the patient had no preexisting metastases/untreated locoregional disease.

Pre-treatment planning CT scans were deformably registered to each subsequent post-treatment scan. The post-treatment scan acted as the template image and the planning CT with its associated PTV and dose map was deformed to this image. Recurrent tumors were contoured on post-treatment scans. Mean dose received by recurrent tumors was recorded using the deformed dose map. The contoured recurrence was compared to the deformed PTV and a category of failure was assigned based on fraction of recurrent tumor falling within the PTV and distance from the closest edge of recurrence to the PTV.

**Figure 1:**
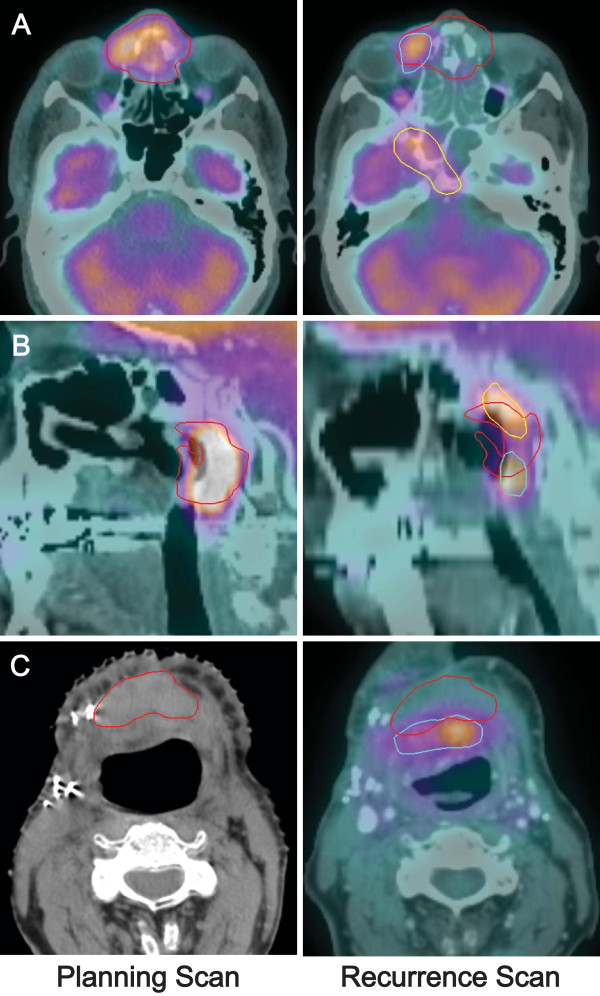
**Examples of categories of failure.** Pre-treatment scans (left) are fused to post-treatment scans demonstrating recurrence (right). Recurrent tumors are contoured, compared to deformed PTVs (red), and categorized. **A**–In-field (blue) and regional (yellow) failure. **B**–Overlap failures (blue, yellow). **C**–Marginal failure (blue).

Previous studies on patterns of failure after Intensity-Modulated Radiation Therapy (IMRT) for primary head and neck cancer have defined in-field recurrences as 95% involvement within clinical target volume (CTV), border-of-field recurrences as 20–95% involvement within CTV, and out-of-field recurrences as below 20% involvement within CTV [21,22]. The current study differs in that we treated recurrent rather than primary head and neck cancer. There are no standard definitions for categories of failure after re-irradiation of recurrent head and neck cancer. Because re-irradiation involves smaller target volumes and minimal margins (no CTV, GTV = PTV), we used a less stringent definition of in-field recurrence. Few local recurrences would otherwise meet a >95% in-field involvement requirement by the time they were detected. We divided the recurrent tumors into four categories of failure: In-field–75% or more of recurrence within PTV, Overlap–20–75% of recurrence within PTV, Marginal–20% or less of recurrence within PTV but closest edge within 1 cm of PTV, Regional/Distant–1 cm or more from PTV. Examples of different patterns of failure are shown in Figure 1.

### Statistical analysis

Endpoints were time to SBRT failure and overall survival. Failure analysis endpoints included time to overall (any) SBRT failure, time to combined In-field/Overlap/Marginal failure (local control), and time to combined Overlap/Marginal failure (near misses). Endpoints were evaluated in both the overall and definitive SBRT cohorts. Multivariate and univariate Cox regressions were used to model predictors of failure and survival. Variables considered in the failure analysis were use of PET-CT planning, tumor volume, use of cetuximab, and time from prior radiation to failure. Variables in the survival analysis included these as well as age, In-field/Overlap/Marginal failure, prior surgery, KPS, and distant metastases/untreated locoregional disease. Variables were entered into the final multivariate model if they reached a significance of p = 0.10. Kaplan-Meier curves were used to estimate time to failure and log-rank tests were used to compare differences between PET-CT planned and non-PET-CT planned patients. SPSS software package was used for statistical analyses [IBM®SPSS® Statistics software Version 19.0].

## Results

### Patient, tumor, and treatment characteristics

Table 1 summarizes patient, tumor, and treatment characteristics. Ninety-six patients with rSCCHN meeting criteria were treated with Cyberknife™ or Trilogy™ SBRT between June 2005 and November 2009. In this cohort, 83% of patients originally presented with stage III–IV primary tumors, with 60% receiving prior chemotherapy, 79% receiving prior surgery, and 48% receiving both. All patients received prior definitive radiation therapy and many received subsequent re-irradiation for pre-SBRT recurrences. Median prior radiation dose was 70 Gy (range, 36–139 Gy). Median time between prior radiation and recurrence was 10 months (range, 1.4–188 months). At the time of SBRT, 40 patients (42%) had other untreated locoregional or distant tumors and were treated palliatively while 56 patients (58%) had no other sites of disease and were treated with curative intent. SBRT was used for positive margins after surgical resection of rSCCHN in seven patients (7%) whereas 89 patients (93%) were treated definitively with SBRT.

**Table 1:** Patient, tumor, and treatment characteristics

**Characteristic**		
Median age (range)	67 yr	(39–91 yr)
Gender:		
Male	70	(73%)
Female	26	(27%)
Primary tumor stage:		
Stage I	4	(4%)
Stage II	6	(6%)
Stage III	19	(20%)
Stage IV	60	(63%)
Unknown	7	(7%)
Prior definitive therapy:		
Radiation only	9	(9%)
Chemo + radiation	11	(12%)
Surgery + radiation	30	(31%)
Chemo, surgery + radiation	46	(48%)
Median prior radiation to head and neck	70 Gy	(36–139 Gy)
Median time from prior radiation to first recurrence	10 mo	(1.4–188 mo)
Median time from prior radiation to SBRT	12 mo	(2.4–179 mo)
Median SBRT dose	44 Gy	(25–50 Gy)
Median tumor volume	26.3 cc	(1–205 cc)
Use of cetuximab with SBRT	41	(43%)
Patients with other distant or untreated tumors	40	(42%)
Patients treated as adjuvant to surgery	7	(7%)
Sites treated with SBRT:		
Oral Cavity	15	(16%)
Nasopharynx	7	(7%)
Oropharynx	13	(14%)
Hypopharynx	7	(7%)
Larynx	7	(7%)
Retropharynx	5	(5%)
Neck	19	(20%)
Base of Skull	10	(11%)
Paranasal Sinuses	5	(5%)
Other	8	(8%)

*Abbreviations*: SBRT = stereotactic body radiation therapy.

Forty-five patients (47%) were planned using PET-CT whereas 51 patients (53%) were planned using non-PET-CT (CT-only: 33 patients, PET-aided CT: 19 patients). Median tumor volume was 26.3 cc (range, 1–205 cc). Median radiation dose was 44 Gy (range, 25–50 Gy) in five fractions and did not differ significantly between the palliatively and curatively treated patients. Forty-six patients (48%) received concurrent biologic agents or chemotherapy with SBRT, with cetuximab being the most common agent (41 patients). All patients completed treatment without toxicity-related breaks and median follow-up was 7.4 months (range, 2.6–52 months)

### Failure assessment

**Figure 2:**
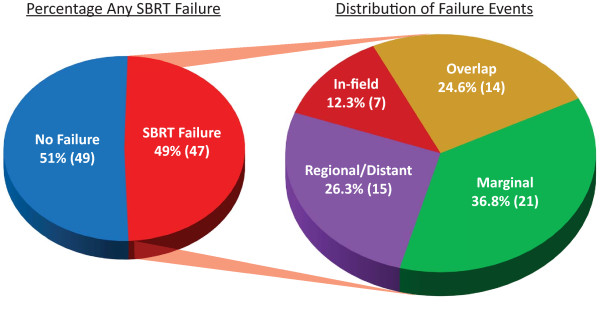
**Distribution of failure outcomes.** 47 out of 96 (49%) patients receiving SBRT for rSCCHN had treatment failures in one or more categories. Distribution of failure events was In-field–7 (12·3%), Overlap–14 (24.6%), Marginal–21 (36.8%), Regional/Distant–15 (26.3%).

SBRT failure outcomes are graphically represented in Figure 2. Of 96 patients, 47 (49%) developed post-SBRT failures. The distribution of failure events was In-field–seven (12.3%), Overlap–14 (24.6%), Marginal–21 (36.8%), Regional/Distant–15 (26.3%). Eleven patients developed two types of failure. Estimated median SBRT failure-free survival was 10.2 months. Among patients that developed post-SBRT failure, median time to failure was 3.8 months. There was no significant difference in prescribed dose for different categories of failure but there was a significance difference in mean dose received within In-field, Overlap, and Marginal failures (44.6 Gy, 37.8 Gy, and 21.2 Gy, respectively, p < 0.001).

Cox regressions are shown in Tables 2, 3, and 4 for time to overall (any) failure, time to In-field/Overlap/Marginal (local) failure, and time to Overlap/Marginal (near miss) failure, respectively. On multivariate analysis of the entire cohort (n = 96), PET-CT treatment planning was associated with longer overall failure-free survival (p = 0.042) and Overlap/Marginal failure-free survival (p = 0.042) than non-PET-CT planning. Analysis of the definitive SBRT subgroup (n = 89) showed a more significant association between PET-CT planning and longer overall failure-free survival (p = 0.011) and Overlap/Marginal failure-free survival (p = 0.009). PET-CT planning was also associated with borderline improvement in In-field/Overlap/Marginal failure-free survival in the definitive SBRT subgroup (p = 0.051). Tumor volume, use of cetuximab, and interval from previous radiation to recurrence were not found to be significant predictors of failure.

**Table 2:** Cox regression analysis for time to Overall (any) failure

	**Overall Cohort (n = 96)**				**Definitive SBRT Subgroup (n = 89)**			
	**Univariate**		**Multivariate**		**Univariate**		**Multivariate**	
	***p***	**HR (95% CI)**	***p***	**HR (95% CI)**	***P***	**HR (95% CI)**	***p***	**HR (95% CI)**
PET-CT Planning	0.042	0.53 (0.29, 0.98)	0.042	0.53 (0.29, 0.98)	0.011	0.45 (0.24, 0.83)	0.011	0.45 (0.24, 0.83)
Tumor volume (5 cc increase)	0.364	1.02 (0.98, 1.06)	0.441	Not entered	0.406	1.02 (0.98, 1.06)	0.517	Not entered
Cetuximab with SBRT	0.916	0.97 (0.54, 1.75)	0.957	Not entered	0.379	0.76 (0.42, 1.39)	0.271	Not entered
Interval from previous radiation to recurrence (3 months increase)								
	0.167	0.98 (0.95, 1.01)	0.188	Not entered	0.221	0.98 (0.95, 1.01)	0.283	Not entered

**Table 3:** Cox regression analysis for time to In field/Overlap/Marginal failure (Local control)

	**Overall Cohort (n = 96)**				**Definitive SBRT Subgroup (n = 89)**			
	**Univariate**		**Multivariate**		**Univariate**		**Multivariate**	
	***p***	**HR (95% CI)**	***p***	**HR (95% CI)**	***p***	**HR (95% CI)**	***p***	**HR (95% CI)**
PET-CT Planning	0.165	0.64 (0.34, 1.20)	0.161	Not entered	0.051	0.53 (0.28, 1.003)	0.051	0.53 (0.28, 1.003)
Tumor volume (5 cc increase)	0.609	1.01 (0.97, 1.05)	0.609	Not entered	0.495	1.003 (0.98, 1.06)	0.568	Not entered
Cetuximab with SBRT	0.861	0.95 (0.51, 1.76)	0.861	Not entered	0.354	0.74 (0.40, 1.39)	0.294	Not entered
Interval from previous radiation to recurrence (3 months increase)								
	0.300	0.99 (0.96, 1.01)	0.297	Not entered	0.361	0.99 (0.96, 1.01)	0.440	Not entered

**Table 4:** Cox regression analysis for time to Overlap/Marginal failure (Near misses)

	**Overall Cohort (n = 96)**				**Definitive SBRT Subgroup (n = 89)**			
	**Univariate**		**Multivariate**		**Univariate**		**Multivariate**	
	***p***	**HR (95% CI)**	***p***	**HR (95% CI)**	***p***	**HR (95% CI)**	***p***	**HR (95% CI)**
PET-CT Planning	0.042	0.48 (0.23, 0.98)	0.042	0.48 (0.23, 0.98)	0.012	0.40 (0.19, 0.82)	0.009	0.40 (0.19, 0.79)
Tumor volume (5 cc increase)	0.415	0.98 (0.92, 1.03)	0.323	Not entered	0.514	0.98 (0.92, 1.04)	0.605	Not entered
Cetuximab with SBRT	0.304	0.70 (0.35, 1.39)	0.337	Not entered	0.089	0.54 (0.27, 1.10)	0.066	0.51 (0.25, 1.04)
Interval from previous radiation to recurrence (3 months increase)								
	0.512	0.99 (0.96, 1.02)	0.604	Not entered	0.596	0.99 (0.96, 1.02)	0.963	Not entered

*Abbreviations*: SBRT = stereotactic body radiation therapy, HR = hazard ratio for failure, CI = confidence interval.

### Impact of planning SIM type on failure

PET-CT treatment planning was found on Cox regression to be a significant predictor of longer failure-free survival. There were no significant differences in age, gender, time from prior radiation to recurrence, prescribed dose, use of cetuximab, tumor differentiation, and tumor volume between the PET-CT and non-PET-CT groups (p > 0.05). A greater number of patients in the PET-CT group had distant metastases/untreated locoregional disease (p = 0.013) but more patients in the non-PET-CT group eventually went on to receive chemotherapy or biologic agents post-SBRT (p = 0.001).

Table 5 shows the frequencies of SBRT failure events and chi-square comparisons. In the overall cohort (n = 96), PET-CT (vs. non-PET-CT) planning was associated with a lower frequency of both overall (38% vs. 59%, p = 0.044) and Overlap/Marginal (24% vs. 47%, p = 0.033) failure. In the definitive SBRT subgroup (n = 89), PET-CT was more strongly associated with a lower rate of both overall (38% vs. 64%, p = 0.020) and Overlap/Marginal (24% vs. 52%, p = 0.009) failure.

**Table 5:** Rates of failure and impact of PET-CT planning vs. non-PET-CT planning on failure

	**Overall Cohort (n = 96)**				**Definitive SBRT Subgroup (n = 89)**				
	**Total**	**PET-CT**	**Non-PET-CT**	***p*** (***X***^**2**^)*	**Total**	**PET-CT**	**Non-PET-CT**	***p*** **(*X***^**2**^)*	
Number of patients:	96	45	51		89	45	44		
Failure vs. No Failure:									
% No Failure (n)	51% (49)	62% (28)	41% (21)	0.044	49% (44)	62% (28)	36% (16)	0.020	
% Overall (Any) Failure (n)	49% (47)	38% (17)	59% (30)	0.044	51% (45)	38% (17)	64% (28)	0.020	
Type of Failure:									
% In-field (n)	7% (7)	11% (5)	4% (2)	0.247	8% (7)	11% (5)	5% (2)	0.434	
% Overlap (n)	15% (14)	11% (5)	18% (9)	0.401	16% (14)	11% (5)	20% (9)	0.258	
% Marginal (n)	22% (21)	13% (6)	29% (15)	0.083	22% (20)	13% (6)	32% (14)	0.045	
% Regional/Distant (n)	16% (15)	13% (6)	18% (9)	0.588	16% (14)	13% (6)	18% (8)	0.573	
Combined Failure Categories:									
% In-field/Overlap/Marginal (n)	43% (41)	33% (15)	51% (26)	0.100	45% (40)	33% (15)	57% (25)	0.034	
% Overlap/Marginal (n)	36% (35)	24% (11)	47% (24)	0.033	38% (34)	24% (11)	52% (23)	0.009	

*Abbreviations*: SBRT = stereotactic body radiation therapy, *Chi-square comparison of PET-CT vs. non-PET-CT planning.

**Figure 3:**
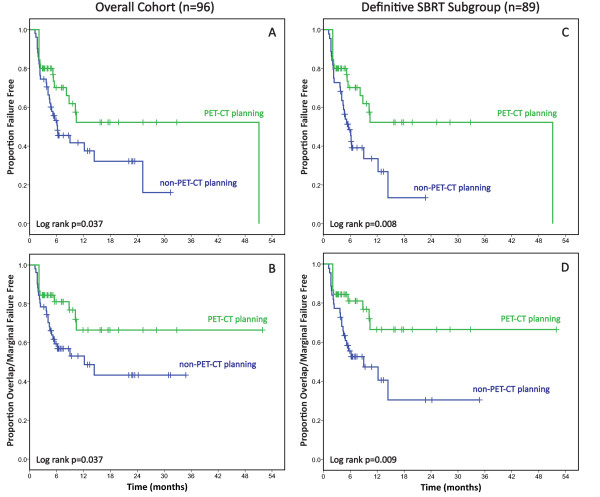
**PET-CT planning vs. non-PET-CT planning.** A,B–Overall failure-free survival **(A)** and Overlap/Marginal failure-free survival **(B)** for PET-CT planning (green) vs. non-PET-CT planning (blue) in the overall cohort (n = 96). C,D–Overall failure-free survival **(C)** and Overlap/Marginal failure-free survival **(D)** for PET-CT planning (green) vs. non-PET-CT planning (blue) in the definitive SBRT cohort (n = 89).

On Kaplan-Meier analysis of PET-CT planning vs. non-PET-CT planning for the overall cohort, there was a significant improvement in time to both overall failure (Figure 3A, p = 0.037) and Overlap/Marginal failure (Figure 3B, p = 0.037) in the PET-CT group. There was no significant difference in time to In-field/Overlap/Marginal failure (p = 0.159). In the definitive SBRT subgroup, PET-CT planning was also associated with a significant increase in time to overall failure and time to Overlap/Marginal failure (Figures 3C, 3D, p = 0.008 and 0.009, respectively). There was a borderline significant increase in time to In-field/Overlap/Marginal failure (p = 0.046).

### Survival assessment

Median overall survival for the entire cohort was 8.2 months. Patients without (vs. patients with) distant metastases/untreated locoregional disease had a significantly longer median overall survival (11.5 months vs. 7.2 months, p = 0.003). Table 6 displays Cox regressions for overall survival. Significant predictors of decreased overall survival on multivariate analysis of the entire cohort (n = 96) were higher tumor volume (p = 0.03), In-field/Overlap/Marginal failure (p = 0.025), older age (p = 0.021), and presence of distant metastases/untreated locoregional disease (p = 0.016). Significant predictors of increased survival were prior surgery (p = 0.004) and higher KPS (p = 0.007). On multivariate analysis of the definitive SBRT cohort (n = 89), tumor volume (p = 0.024), age (p = 0.012), and presence of distant metastases/untreated locoregional disease (p = 0.037) remained significant predictors of decreased overall survival. Prior surgery (p = 0.01) and KPS (p = 0.028) remained significant predictors of increased survival. PET-CT planning, use of cetuximab, and interval from prior radiation to recurrence were not significant predictors of survival.

**Table 6:** Cox regression analysis for overall survival

	**Overall Cohort (n = 96)**				**Definitive SBRT Subgroup (n = 89)**			
	**Univariate**		**Multivariate**		**Univariate**		**Multivariate**	
	***p***	**HR (95% CI)**	***p***	**HR (95% CI)**	***p***	**HR (95% CI)**	***p***	**HR (95% CI)**
PET-CT Planning	0.640	1.1 (0.72, 1.71)	0.708	Not entered	0.657	0.91 (0.58, 1.41)	0.960	Not entered
Tumor volume (5 cc increase)	0.036	1.03 (1.001, 1.06)	0.030	1.04 (1.006, 1.07)	0.025	1.03 (1.001, 1.06)	0.024	1.04 (1.006, 1.07)
Cetuximab with SBRT	0.192	1.36 (0.86, 2.14)	0.431	Not entered	0.700	1.10 (0.69, 1.74)	0.827	Not entered
Interval from previous radiation to recurrence (3 month increase)								
	0.731	0.997 (0.97, 1.02)	0.475	Not entered	0.845	0.997 (0.98, 1.01)	0.433	Not entered
Any In-field/Overlap/Marginal failure								
	0.141	1.39 (0.90, 2.15)	0.025	1.71 (1.07, 2.74)	0.547	1.15 (0.74, 1.78)	0.095	1.50 (0.93, 2.43)
Age (10 year increase)	0.273	1.11 (0.93, 1.32)	0.021	1.25 (1.04, 1.49)	0.128	1.15 (0.96, 1.37)	0.012	1.27 (1.05, 1.54)
Any Prior Surgery	0.007	0.50 (0.30, 0.83)	0.004	0.46 (0.27, 0.78)	0.023	0.55 (0.33, 0.92)	0.010	0.49 (0.29, 0.85)
KPS (10 KPS increase)	0.010	0.80 (0.69, 0.94)	0.007	0.79 (0.66, 0.94)	0.041	0.84 (0.71, 0.99)	0.028	0.82 (0.69, 0.98)
Any distant metastases or untreated locoregional disease								
	0.004	1.95 (1.24, 3.07)	0.016	1.80 (1.12, 2.90)	0.024	1.69 (1.07, 2.67)	0.037	1.66 (1.03, 2.69)

*Abbreviations*: SBRT = stereotactic body radiation therapy, HR = hazard ratio for death, CI = confidence interval, KPS = Karnofsky performance status.

## Discussion

Interest in SBRT for rSCCHN has grown due to its ability to deliver high doses of radiation to focal areas with high precision, potentially reducing toxicity and shortening treatment courses [9-12]. This study serves as the first analysis of patterns of failure after SBRT for rSCCHN, and thus provides crucial information for advancing the field and enhancing efficacy of this novel targeted therapy. The availability of many bony landmarks in the head and neck enhances accuracy of deformable registration and allows a detailed pattern of failure analysis. Our failure categories were, in order of proximity to the PTV: In-field, Overlap, Marginal, and Regional/Distant. We found that most failures were on or near PTV borders (Overlap/Marginal failure, 61.4%), rather than within the PTV (In-field failure, 12.3%) or over 1 cm from the PTV (Regional/Distant failure, 26.3%).

Whereas most prior pattern of failure analyses of radiation for primary head and neck SCC showed mostly in-field recurrences [21,22], we found a high frequency of edge-of-field failures after SBRT for rSCCHN. Our findings also stand in contrast to one of the few other existing studies on patterns of failure after re-irradiation of rSCCHN, which used IMRT and 3-D conformal radiation therapy rather than SBRT. In that study, Popovtzer et al. reported a large majority of in-field recurrences and suggested that confining radiation targets to the GTV could reduce toxicity without compromising overall control [23]. However, that study used a 5 mm margin around the GTV (vs. no margin in our study) and a less stringent definition of in-field recurrence (>50% of recurrence within 95% isodose curve vs. >75% of recurrence within PTV in our study), possibly resulting in a lower frequency of near miss failures. When we applied Popovtzer et al.’s definitions to our database, Overlap/Marginal failures still accounted for 53% of failures.

Treatment of recurrent head and neck cancer differs from primary tumors in that for recurrences, prophylactic contouring of regions of lymphatic drainage and large margins are not standard.^8^ With the smaller targets and the sharp dose fall-off involved in SBRT for rSCCHN, the potential for near misses increases and accurate detection and contouring of tumor edges becomes critical. Detection of tumor edges may be enhanced with PET-CT treatment planning. We found PET-CT planning to be associated with longer overall failure-free survival and longer Overlap/Marginal failure-free survival after SBRT for rSCCHN, suggesting an advantage in tumor control. This association was stronger in the definitive SBRT subgroup, which likely reflects the higher usage of CT planning in adjuvant treatments for positive surgical margins included in the overall cohort. For time to combined In-field/Overlap/Marginal failure, PET-CT showed no significant benefit in the overall cohort and only a borderline benefit in the definitive SBRT subgroup. This suggests that PET-CT planning improves control principally through reduction of failures on the PTV edge.

Overall survival for patients without distant metastases or untreated locoregional disease in this study was similar to prior reports on SBRT for rSCCHN [9-12]. We found that older age, lower performance status (KPS), presence of distant metastases/untreated locoregional disease, higher tumor volume, and lack of prior surgery were significant predictors of decreased overall survival. We did not observe a significant difference in overall survival between the PET-CT and non-PET-CT planning groups.

A primary limitation of the current study is our inability to detect precisely where recurrent tumors originated. Recurrent tumors are often large by the time they are detected and if any part of the recurrence overlaps with the PTV border, clinical judgment becomes important in defining whether the recurrence originated from inside or outside the PTV. This may lead to subjective categorization of failures as in-field vs. out of field and subsequent variation in definitions of category of failure. Other limitations include patient heterogeneity and potential variability in contouring recurrent tumors.

## Conclusions

This study demonstrates that most recurrences after SBRT for rSCCHN were near misses, i.e. failures occurring near the PTV border. PET-CT treatment planning was shown to result in lower rates of failure, particularly near miss failures, compared to non-PET-CT planning. PET-CT appears advantageous for treatment planning in SBRT for rSCCHN, where accurate targeting of smaller tumor volumes within previously-irradiated tissues is critical.

## Competing interests

We declare that we have no competing interests. Funding was provided by the Department of Radiation Oncology, University of Pittsburgh Cancer Institute.

## Authors’ contributions

DEH designed and had oversight of the study. KW collected data, performed statistical analyses, and wrote the manuscript. JCF and JMR helped with statistical analyses. CO helped with software. All other authors reviewed, edited, and approved the final manuscript.
